# Effects of organic zinc on the performance and gut integrity of broilers under heat stress conditions

**DOI:** 10.5194/aab-63-125-2020

**Published:** 2020-04-27

**Authors:** Mohannad Abuajamieh, Anas Abdelqader, Rabie Irshaid, Firas M. F. Hayajneh, Ja'far M. Al-Khaza'leh, Abdur-Rahman Al-Fataftah

**Affiliations:** 1 Department of Animal Production, School of Agriculture, The University of Jordan, Amman 11942, Jordan; 2 Department of Animal Production, Faculty of Agricultural Technology, Al-Balqa Applied University, Al-Salt 19117, Jordan

## Abstract

Heat stress (HS) has negative impacts on farm animals. Many studies have been conducted in order to ameliorate the effects of heat stress in farm animals. The current project investigated the effects of organic zinc supplementation under thermoneutral and heat stress conditions on the production, physiological, and histological parameters in broiler chickens. Three-hundred and sixty chicks in the current project were assigned randomly to six different treatments (n=60 chicks per treatment). The treatments were (1) a basal diet containing 40 mg kg${}^{-1}$ of Zn from an organic source and rearing under thermoneutral (TN) conditions (Ctrl); (2) a diet containing the amount of Zn from the basal diet +50 % of the Zn level (from the basal diet) and rearing under TN conditions (50 TN); (3) a diet containing the amount of Zn from the basal diet +100 % of the Zn level (from the basal diet) and rearing under TN conditions (100 TN); (4) a basal diet containing 40 mg kg${}^{-1}$ of Zn from an organic source and exposure to 3 d of cyclical HS at the age of 35 d (CHS); (5) a diet containing the amount of Zn from the basal diet +50 % of the Zn level (from the basal diet) and exposure to 3 d of cyclical HS at the age of 35 d (50 HS); and (6) a diet containing the amount of Zn from the basal diet +100 % of the Zn level (from the basal diet) and exposure to 3 d of cyclical HS at the age of 35 d (100 HS). Our results indicated that HS has decreased final body weight (fBW), average daily gain (ADG), and feed conversion ratio (FCR) relative to TN chicks. However, organic zinc had little or no effects on the production parameters measures in the current project. Overall, intestinal histological measurements were negatively altered under HS relative to TN chicks. Organic zinc inclusion in the diet had improved villus height in the duodenum and jejunum relative to the Ctrl and CHS chicks. Blood calcium and glucose levels were decreased and increased, respectively, in HS relative to TN chicks. In summary, the results discussed in the current project revealed that the inclusion rates of organic zinc used here had little or no effects on the productive parameters. However, it improved the morphological characteristics of the intestines which might maximized the intestinal efficiency in nutrient absorption under HS conditions.

## 1 Introduction

Heat stress (HS) compromises the overall performance of farm animals. In Jordan, the temperatures in summer increase beyond the upper critical temperature for livestock species and poultry, which decreased their performance and leads to high mortality in severe heat waves. The reduction in animals performance is reflected in the profitability of the farmers especially, the smallholders. Thus, there is an urgent and continuous need to investigate new management strategies to overcome the effects of heat stress.

In the last few decades, the negative effects of heat stress on the performance of farm animals has become more severe. Some reports have indicated that the Earth's temperature might increase in the future (Luber and McGeehin, 2008). In fact, Pachauri and Meyer (2014) expected the ambient temperature to increase by more than 5${}^{\circ }$ by 2100.

High summer temperatures along with sudden heat waves have huge negative effects on the smallholdings where the heat abatement strategies taken are at minimum. Heat-stress-related problems and heat waves will become more frequent in the future due to global warming (Horton et al., 2016). In Jordan, summer months have high ambient temperatures, which might exceed 45 ${}^{\circ }$C, according to the report issued by the department of Jordanian meteorology in 2014; in the Jordan Valley the summer temperature reached 50 ${}^{\circ }$C. Moreover, in August of 2015, Amman (the capital) was hit by a heat wave for 9 consecutive days (the maximum and minimum temperatures recorded by the University of Jordan meteorological station were 38 and 22 ${}^{\circ }$C, respectively). Thus, heat stress increases the economic losses of poultry and farm animal mortality in addition to the reduction in production quantity and quality if heat abatement strategies are not developed urgently.

The current project investigated the effects of organic zinc supplementation under heat stress on the production, physiological, and histological parameters in broiler chickens. Zinc is intensively used by the farmers in summer months as a dietary approach to alleviate the negative impacts of heat stress and to improve productivity (Manner and Wang, 1991; Chand et al., 2014). However, the responses of animals to dietary minerals depend on many factors such as animal species, inclusion rate, form, availability, and age of the animals. Zinc is known to have pivotal roles for more than 300 enzymes and more than 2000 transcription factors (Swecker, 2014). Zinc is also involved in the normal function of the immune system and skeletal muscles development and has an antioxidant role (Sahin et al., 2009). Some studies have reported that organic zinc is also more bioavailable when compared with inorganic zinc (Wedekind et al., 1992; Rabiee et al., 2010).

Many researches have reported the beneficiary effects of supplementing zinc under normal conditions (Overton and Yasui, 2014; Eze et al., 2015). To our knowledge, no previous studies have been conducted to investigate the effects of organic zinc supplementation in the finisher phase on poultry under cyclic heat stress, and so investigating the effects of organic zinc in the finisher phase on broiler chickens under cyclic heat stress conditions may provide a practical tool and novel findings to alleviate the heat stress negative impacts, improve performance, and reduce heat-stress-associated mortality. Therefore, the main objectives of the current project were to study the effects of organic zinc supplemented to the finisher diet on poultry under heat stress conditions and to investigate the potential of organic zinc to mitigate the heat-stress-associated problems in order to enhance chicken performance.

## 2 Material and methods

### 2.1 Animals and experimental design

**Figure 1:**
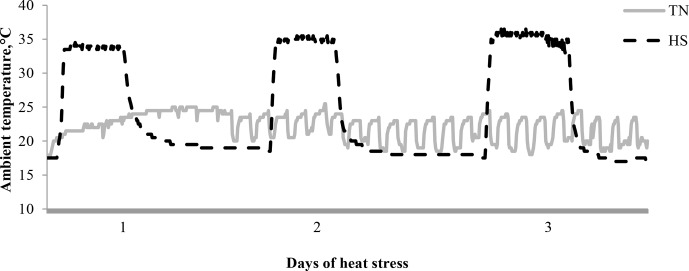
Ambient temperature (Ta; ${}^{\circ }$C) by day of heat stress. Abbreviations are as follows: thermoneutral environment (TN; 22.6±1.8 ${}^{\circ }$C) and heat stress environment (HS; cyclical 19.8±1.1 ${}^{\circ }$C, from 15:00 to 11:00; 33.7±0.6 ${}^{\circ }$C from 11:00 to 15:00 LT).

A total of 360 Ross 308 classic chicks (1 d old) with an average body weight (BW) of 42.5 g ± 3 were purchased from a commercial hatchery and utilized in the current experiment. Upon arrival to the Animal Physiology Lab (The University of Jordan), the chicks were brooded in electrical battery cages and provided with the required temperature, feed, and water. At 21 d of age, chicks were randomly assigned to six treatments; each treatment had six replicates (n=10 chicks/replicate). Chicks were assigned to one of the following treatments: (1) a diet containing 40 mg kg${}^{-1}$ of Zn from an organic source (Availa Zn^®^, Zinpro Corporation) +80 mg kg${}^{-1}$ of Zn from a mineral premix (a total of 120 mg kg${}^{-1}$ of Zn) and rearing under thermoneutral (TN) conditions (Ctrl; n=60); (2) a diet containing 60 mg kg${}^{-1}$ of Zn from an organic source (+50 % of the organic Zn level from the Ctrl treatment) +80 mg kg${}^{-1}$ of Zn from a mineral premix (a total of 140 mg kg${}^{-1}$ of Zn) and rearing under TN conditions (50 TN; n=60); (3) a diet containing 80 mg kg${}^{-1}$ of Zn from an organic source (+100 % of the organic Zn level from the Ctrl treatment) +80 mg kg${}^{-1}$ of Zn from a mineral premix (a total of 160 mg kg${}^{-1}$ of Zn) and rearing under TN conditions (100 TN; n=60); (4) a diet containing 40 mg kg${}^{-1}$ of Zn from an organic source + 80 mg kg${}^{-1}$ of Zn from a mineral premix (a total of 120 mg kg${}^{-1}$ of Zn) and exposure to cyclical heat stress (HS) at the age of 35 d for 3 consecutive days (CHS; n=60); (5) a diet containing 60 mg kg${}^{-1}$ of Zn from an organic source (+50 % of the organic Zn level from the Ctrl treatment) +80 mg kg${}^{-1}$ of Zn from a mineral premix (a total of 140 mg kg${}^{-1}$ of Zn) and exposure to cyclical HS at the age of 35 d for 3 consecutive days (50 HS; n=60); and (6) a diet containing 80 mg kg${}^{-1}$ of Zn from an organic source (+100 % of the organic Zn level from the Ctrl treatment) +80 mg kg${}^{-1}$ of Zn from a mineral premix (a total of 160 mg kg${}^{-1}$ of Zn) and exposure to cyclical HS at the age of 35 d for 3 consecutive days (100 HS; n=60). The level of Zn in the Ctrl diet was similar to the Zn level used in other studies (Moghaddam and Jahanian, 2009; Jahanian and Rasouli, 2015) and the recommended Zn level for broilers (Council, 1994). All procedures were approved by the University of Jordan Animal Care and Use Committee. The ambient temperatures of the TN and HS conditions are shown in Fig. 1.

**Table 1:** Ingredients and composition of nutrients.

Ingredient, %	Starter	Grower	Finisher
	diet	diet	diet
Corn grain	56 000	61 000	64 800
Soybean meal (48 % CP)	39 300	32 800	28 800
Vegetable oil	2000	3500	3700
Mono-calcium phosphate	1200	1000	1000
DL-methionine (98 %)	0.320	0.280	0.280
L-lysine-HCL (98.5 %)	0.300	0.270	0.270
Threonine	0.080	0.080	0.080
NaCl	0.200	0.200	0.200
Vitamin premix${}^{1}$	0.100	0.100	0.100
Mineral premix${}^{2}$	0.100	0.100	0.100
Choline chloride (70 %)	0.080	0.080	0.080
Coccidiostat	0.050	0.050	0.050
Concentrate 2.5 %	0.270	0.540	0.540
Nutrient chemical composition			
ME kcal kg${}^{-1}$ feed	3000	3100	3150
Crude protein (CP), %	23.0	19.0	18.0
Methionine, %	0.65	0.50	0.45
Lysine, %	1.50	1.35	1.15
Threonine, %	1.00	0.85	0.70
Tryptophan, %	0.31	0.28	0.25
Calcium, %	1.10	1.00	0.95
Phosphorous, %	0.55	0.50	0.50

${}^{1}$ Vitamin premix provided the following (per kg diet): 120 000 IU of vitamin A, 3500 IU of vitamin D${}_{3}$, 40 IU of vitamin E, 2.5 mg of vitamin B${}_{1}$, 8 mg of vitamin B${}_{2}$, 5 mg of vitamin B${}_{6}$, 150 µg of riboflavin, 30 µg of B${}_{12}$, 1.5 mg of folic acid, 45 mg of niacin, and 13 mg of pantothenic acid. ${}^{2}$ Mineral premix provided the following (per kg diet): 60 mg of Fe, 10 mg of Cu, 80 mg of Mn, 80 mg of Zn, 1 mg of I, and 0.2 mg of Se.

### 2.2 Diet mixing

During the whole experiment, diets were prepared at Al-Estesharia for Poultry and Feed Co. Amman. The diet ingredients and composition are illustrated in Table 1. Different bag colors were used to allow for visual differentiation between the three diets in an attempt to prevent mixing errors. The broiler diets were formulated to meet or exceed the nutrient and energy predicted requirements (Council, 1994)

### 2.3 Data collection

The data in the current experiment were collected in two phases: the feeding phase and the HS phase. The feeding phase of the experiment lasted for 14 d (22 to 35 d of age) where chicks were fed their respective diets ad libitum and feed intake and body weights were recorded on a daily and weekly basis, respectively. Feed was provided daily at 08:00 LT and orts were recorded daily prior to the AM feeding. During the feeding phase, all chicks were under TN conditions (22.6±1.8 ${}^{\circ }$C; 52±7 % relative humidity, RH). Following the feeding phase, chicks of the HS treatments (HS, 50 HS, and 100 HS) were moved into an environmentally controlled chamber (at the age of 36 d) to conduct the HS phase. The HS phase lasted for 3 consecutive days (days 36 to 38); heat stress conditions were cyclical and consisted of 19.8±1.1 ${}^{\circ }$C and 45.3 % relative humidity, from 15:00 to 11:00 LT, 33.7±0.6 ${}^{\circ }$C and 40.9 % RH, from 11:00 to 15:00 LT (Fig. 1). Ambient temperature was controlled, but humidity was not governed, and both parameters were recorded every 5 min by a data logger (Lascar EL-USB-2-LCD, Erie, PA) in the chamber.

### 2.4 Rectal temperature ($T_{r}$)

During HS phase, $T_{r}$ were measured twice a day (after the third and fourth hours of HS) in all treatments using a standard digital thermometer (GLA M700 Digital Thermometer, San Luis Obispo, CA).

### 2.5 Blood parameters

At sacrifice (day 39), fresh blood samples were collected from the wing vein and assayed immediately using an epoc^®^ blood analysis system (Siemens Healthineers; GmbH, Germany), which measured blood glucose and ionized calcium (iCa).

### 2.6 Postmortem tissue collection

On day 39, chicks were euthanized by cutting the jugular vein. Organs and tissues were harvested immediately after sacrifice. Liver, heart, spleen, and intestine weights were recorded. Intestinal tissues were harvested within 5 min following euthanasia. The duodenum was collected 5 cm distal to the pyloric sphincter. The jejunum was collected at 15 cm before the Meckel's diverticulum. The ileum was collected at 15 cm after the Meckel's diverticulum. All the intestinal segments (10–20 cm, approximately) were flushed with cold phosphate buffer saline to remove intestinal content and fixed in 10 % neutral buffered formalin for later histological analysis.

### 2.7 Intestinal histology

For histological analysis, formalin-fixed duodenum, jejunum, and ileum samples were submitted to the Histology Lab at the School of Medicine, University of Jordan, within 1 week of euthanasia for sectioning and periodic acid–Schiff (PAS) staining. Two chicks were chosen from each replicate to obtain intestinal samples (12 chicks per treatment). Three slides per chick per tissue were generated. Using a microscope (Leica DM750 microscope) with an attached camera, five images per section of intestine were obtained at 100× magnification. All image processing and quantification was done using Leica LAS EZ 3.4 software. Results of the five images per intestinal section were condensed into a single average per chick.

Villus height was measured from the tip to the level of the villus–crypt interface utilizing the segmented line tool along the villus midline. Villus width was measured using a single line at mid-villus height. Crypt depth was measured with a single line from the villus–crypt interface to the laminae propria and muscularis mucosa junction. A mucosal surface area estimate was obtained using the mucosal-to-serosal amplification ratio M, as previously reported by Kisielinski et al. (2002), where

1 $$\begin{array}{rl} & M=\\ & \frac{\left(\text{villus width}\;\times \;\text{villus length}\right)+{\left(\frac{\text{villus width}}{2}+\frac{\text{crypt width}}{2}\right)}^{2}-{\left(\frac{\text{villus width}}{2}\right)}^{2}}{{\left(\frac{\text{villus width}}{2}+\frac{\text{crypt width}}{2}\right)}^{2}} \end{array}.$$

### 2.8 Statistical analysis

The effect of treatment (Ctrl, 50 TN, 100 TN, CHS, 50 HS, and 100 HS) on variables with single measures was analyzed using PROC MIXED of SAS (9.4 Inst. Inc., Cary, NC). For variables with multiple measurements over time, a repeated measures analysis with an autoregressive covariance structure and day as the repeated effect was used to determine the effects of treatment, day, and treatment–day interaction. For both single and repeated measure variables, preplanned contrasts were evaluated: TN vs. HS, TN vs. TN, and HS vs. HS using the CONTRAST statement of SAS. Additional contrasts were done for specific treatments or their combinations when relevant and their P values are reported if significant. For each variable, the residuals' distribution was tested for normality, and logarithmic transformation was performed when necessary. Results are reported as least squares means and considered different when P≤0.05 and tend to differ if P<0.10.

**Figure 2:**
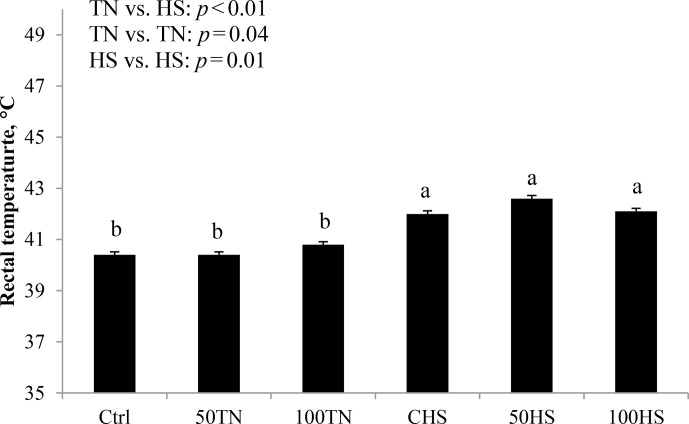
Effects of feeding different levels of organic zinc under TN and HS conditions on rectal temperature in broiler chickens. Results are expressed as the least squares mean (LSM) ± standard error of the mean (SEM).

**Table 2:** Effects of feeding different levels of organic zinc under TN and HS conditions on BW, ADFI, ADG, FCR, and DP in broiler chickens.

Parameter	Treatments						P value	Contrasts		
	Ctrl${}^{1}$	50 TN${}^{2}$	100 TN${}^{3}$	CHS${}^{4}$	50 HS${}^{5}$	100 HS${}^{6}$	Treatment	TN vs. HS	TN vs. TN	HS vs. HS
iBW, g${}^{7}$	889.3 ± 15.0	879.5 ± 17.3	895.2 ± 10.1	867.1 ± 5.2	857.5 ± 17.3	899.9 ± 8.2	0.21	0.59	0.40	0.25
fBW, g${}^{8}$	2048 ± 20${}^{\text{a}}$	1998 ± 29${}^{\text{a}}$	2017 ± 19${}^{\text{a}}$	2044 ± 11${}^{\text{a}}$	1897 ± 18${}^{\text{b}}$	2006 ± 13${}^{\text{a}}$	<0.01	0.02	0.49	<0.01
ADFI, grams per day${}^{9}$	157.7 ± 1.5	159.9 ± 3.2	154.0 ± 1.0	159.6 ± 1.8	155.7 ± 2.0	153.3 ± 2.7	0.16	0.58	0.06	0.42
ADG, grams per day${}^{10}$	83.0 ± 1.5${}^{\text{ab}}$	79.9 ± 1.4${}^{\text{bc}}$	80.1 ± 1.0${}^{\text{bc}}$	83.8 ± 0.9${}^{\text{a}}$	74.2 ± 0.7${}^{\text{d}}$	79.0 ± 1.1${}^{\text{c}}$	<0.01	0.04	0.88	<0.01
FCR (g kg${}^{-1}$)${}^{11}$	1.90 ± 0.02${}^{\text{c}}$	2.00 ± 0.02${}^{\text{b}}$	1.92 ± 0.02${}^{\text{c}}$	1.90 ± 0.03${}^{\text{c}}$	2.10 ± 0.02${}^{\text{a}}$	1.94 ± 0.01${}^{\text{c}}$	<0.01	0.03	<0.01	<0.01
DP${}^{12}$	60.7 ± 0.4${}^{\text{y}}$	59.4 ± 0.7${}^{\text{y}}$	60.3 ± 0.2${}^{\text{y}}$	61.4 ± 0.5${}^{\text{xy}}$	59.8 ± 1.2${}^{\text{y}}$	62.4 ± 0.4${}^{\text{x}}$	0.08	0.08	0.68	<0.01

${}^{1}$ Control chicks under thermoneutral conditions (22.6 ± 1.8 ${}^{\circ }$C; 52 ± 7 % RH) that received 40 ppm of organic Zn. ${}^{2}$ Chicks under thermoneutral conditions (22.6 ± 1.8 ${}^{\circ }$C; 52 ± 7 % RH) supplemented with a diet containing the amount of Zn from the basal diet +50 % of the Zn from the basal diet. ${}^{3}$ Chicks under thermoneutral conditions (22.6 ± 1.8 ${}^{\circ }$C; 52 ± 7 % RH) supplemented with a diet containing the amount of Zn from the basal diet +100 % of the Zn from the basal diet. ${}^{4}$ Chicks exposed to cyclical heat stress (19.8 ± 1.1 ${}^{\circ }$C, 45.3 % relative humidity, from 15:00 to 11:00 LT; 33.7 ± 0.6 ${}^{\circ }$C, 40.9 % RH, from 11:00 to 15:00 LT) on days 36–38 that received 40 ppm of organic Zn. ${}^{5}$ Chicks exposed to cyclical heat stress (19.8 ± 1.1 ${}^{\circ }$C, 45.3 % relative humidity, from 15:00 to 11:00 LT; 33.7 ± 0.6 ${}^{\circ }$C, 40.9 % RH, from 11:00 to 15:00 LT) on days 36–38 supplemented with a diet containing the amount of Zn from the basal diet +50 % of the Zn from the basal diet. ${}^{6}$ Chicks exposed to cyclical heat stress (19.8 ± 1.1 ${}^{\circ }$C, 45.3 % relative humidity, from 15:00 to 11:00 LT; 33.7 ± 0.6 ${}^{\circ }$C, 40.9 % RH, from 11:00 to 15:00 LT) on days 36–38 supplemented with a diet containing the amount of Zn from the basal diet +50 % of the Zn from the basal diet. ${}^{7}$ Initial body weight at day 21. ${}^{8}$ Body weight at the day of euthanasia (day 39). ${}^{9}$ Average daily feed intake. ${}^{10}$ Average daily gain. ${}^{11}$ Feed conversion ratio. ${}^{12}$ Dressing percentage ((carcass weight/live weight) × 100). ${}^{\text{a,b,c}}$ P≤0.05. ${}^{\text{x,y}}$ 0.05<P≤0.10.

## 3 Results

### 3.1 Performance parameters

During the heat stress challenge, $T_{r}$ was markedly increased in HS-exposed chicks (1.7 ${}^{\circ }$C; P<0.01; Fig. 2) compared to TN chicks, which confirms that HS was successfully implemented in the current project. Initial BW (day 21) was similar in all treatments (P=0.21; Table 2). However, final BW (fBW) was increased in TN relative to the HS chicks (2 %; P=0.02; Table 2). Regardless of the Zn inclusion rate, there was a similar average daily feed intake (ADFI) for both TN and HS chicks (Table 2). Overall, HS chicks had a slightly decreased average daily gain (ADG; 2.4 %; P=0.04; Table 2) compared to TN chicks. On the other hand, the feed conversion ratio (FCR) was increased in HS chicks when compared to TN chicks (2 %; P=0.03; Table 2). Interestingly, CHS and 100 HS FCR were similar to the FCR in Ctrl chicks (Table 2). In addition, HS chicks tended to have increased dressing percentage (DP; ∼2 %; P=0.08; Table 2) compared with TN chicks.

**Figure 3:**
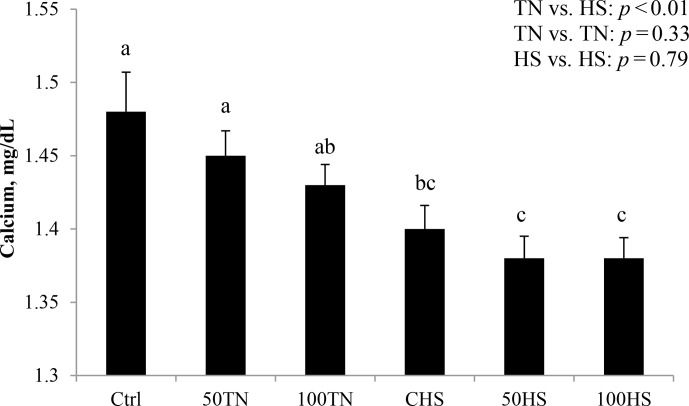
Effects of feeding different levels of organic zinc under TN and HS conditions on blood calcium in broiler chickens. Results are expressed as LSM ± SEM.

### 3.2 Blood ionized calcium and glucose

**Figure 4:**
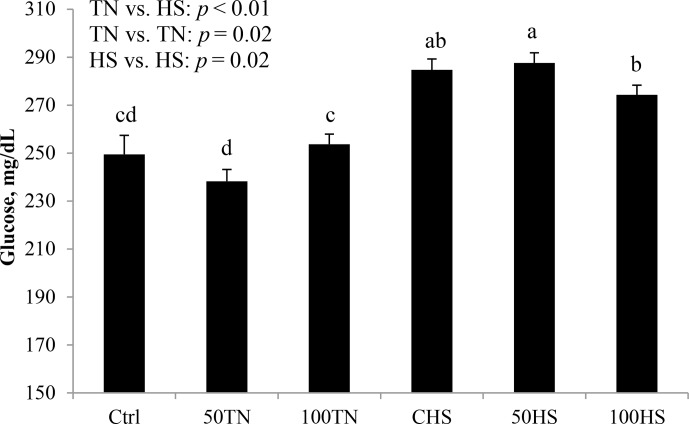
Effects of feeding different levels of organic zinc under TN and HS conditions on blood glucose in broiler chickens. Results are expressed as LSM ± SEM.

Blood ionized calcium decreased in HS relative to TN chicks (4 %; P<0.01; Fig. 3). Relative to TN chicks, blood glucose levels in HS chicks measured directly after euthanasia were increased (16 %; P<0.01; Fig. 4).

**Table 3:** Effects of feeding different levels of organic zinc under TN and HS conditions on liver, abdominal fat, heart, intestines, and spleen measurements postmortem.

Parameter	Treatments						P value	Contrasts		
	Ctrl${}^{1}$	50 TN${}^{2}$	100 TN${}^{3}$	CHS${}^{4}$	50 HS${}^{5}$	100 HS${}^{6}$	Treatment	TN vs. HS	TN vs. TN	HS vs. HS
Liver										
Weight (g)	58.2 ± 2.3${}^{\text{ab}}$	62.6 ± 3.3${}^{\text{a}}$	57.0 ± 1.7${}^{\text{ab}}$	50.3 ± 1.3${}^{\text{c}}$	59.2 ± 1.6${}^{\text{ab}}$	55.3 ± 2.2${}^{\text{bc}}$	<0.01	0.02	0.08	0.21
% of BW	2.37 ± 0.05${}^{\text{ab}}$	2.58 ± 0.11${}^{\text{a}}$	2.57 ± 0.10${}^{\text{a}}$	2.21 ± 0.06${}^{\text{b}}$	2.58 ± 0.04${}^{\text{a}}$	2.32 ± 0.10${}^{\text{b}}$	<0.01	0.05	0.90	0.03
Abdominal fat										
Weight (g)	36.9 ± 1.7${}^{\text{a}}$	34.5 ± 2.8${}^{\text{a}}$	25.6 ± 2.5${}^{\text{b}}$	35.5 ± 1.8${}^{\text{a}}$	26.7 ± 2.6${}^{\text{b}}$	23.9 ± 1.6${}^{\text{b}}$	<0.01	0.05	<0.01	0.39
% of BW	1.52 ± 0.08${}^{\text{a}}$	1.44 ± 0.12${}^{\text{ab}}$	1.14 ± 0.10${}^{\text{c}}$	1.55 ± 0.09${}^{\text{a}}$	1.17 ± 0.12${}^{\text{bc}}$	1.00 ± 0.06${}^{\text{c}}$	<0.01	0.14	0.03	0.22
Heart										
Weight (g)	12.2 ± 0.5	11.9 ± 0.3	11.9 ± 0.6	11.5 ± 0.3	11.9 ± 0.4	12.1 ± 0.5	0.93	0.61	0.95	0.84
% of BW	0.50 ± 0.01	0.49 ± 0.01	0.53 ± 0.02	0.50 ± 0.01	0.52 ± 0.02	0.50 ± 0.02	0.63	0.85	0.13	0.47
Intestines										
Weight (g)	89.3 ± 2.0${}^{\text{x}}$	88.7 ± 2.2${}^{\text{x}}$	81.2 ± 5.4${}^{\text{xy}}$	78.2 ± 3.8${}^{\text{y}}$	82.1 ± 0.9${}^{\text{xy}}$	80.8 ± 2.6${}^{\text{xy}}$	0.08	0.02	0.10	0.77
% of BW	3.71 ± 0.18	3.68 ± 0.08	3.55 ± 0.15	3.42 ± 0.16	3.60 ± 0.09	3.36 ± 0.05	0.33	0.08	0.46	0.19
Spleen										
Weight (g)	2.42 ± 0.27${}^{\text{ab}}$	2.20 ± 0.18${}^{\text{b}}$	2.05 ± 0.16${}^{\text{b}}$	2.08 ± 0.16${}^{\text{b}}$	2.95 ± 0.23${}^{\text{a}}$	2.18 ± 0.17${}^{\text{b}}$	0.02	0.28	059	<0.01
% of BW	0.097 ± 0.009${}^{\text{b}}$	0.093 ± 0.009${}^{\text{b}}$	0.093 ± 0.008${}^{\text{b}}$	0.090 ± 0.006${}^{\text{b}}$	0.127 ± 0.009${}^{\text{a}}$	0.090 ± 0.007${}^{\text{b}}$	0.01	0.22	0.99	<0.01

${}^{1}$ Control chicks under thermoneutral conditions (22.6 ± 1.8 ${}^{\circ }$C; 52 ± 7 % RH) that received 40 ppm of organic Zn. ${}^{2}$ Chicks under thermoneutral conditions (22.6 ± 1.8 ${}^{\circ }$C; 52 ± 7 % RH) supplemented with a diet containing the amount of Zn from the basal diet +50 % of the Zn from the basal diet. ${}^{3}$ Chicks under thermoneutral conditions (22.6 ± 1.8 ${}^{\circ }$C; 52 ± 7 % RH) supplemented with a diet containing the amount of Zn from the basal diet +100 % of the Zn from the basal diet. ${}^{4}$ Chicks exposed to cyclical heat stress (19.8 ± 1.1 ${}^{\circ }$C, 45.3 % relative humidity, from 15:00 to 11:00 LT; 33.7 ± 0.6 ${}^{\circ }$C, 40.9 % RH, from 11:00 to 15:00 LT) on days 36–38 that received 40 ppm of organic Zn. ${}^{5}$ Chicks exposed to cyclical heat stress (19.8 ± 1.1 ${}^{\circ }$C, 45.3 % relative humidity, from 15:00 to 11:00 LT; 33.7 ± 0.6 ${}^{\circ }$C, 40.9 % RH, from 11:00 to 15:00 LT) on days 36–38 supplemented with a diet containing the amount of Zn from the basal diet +50 % of the Zn from the basal diet. ${}^{6}$ Chicks exposed to cyclical heat stress (19.8 ± 1.1 ${}^{\circ }$C, 45.3 % relative humidity, from 15:00 to 11:00 LT; 33.7 ± 0.6 ${}^{\circ }$C, 40.9 % RH, from 11:00 to 15:00 LT) on days 36–38 supplemented with a diet containing the amount of Zn from the basal diet +50 % of the Zn from the basal diet. ${}^{\text{a,b,c}}$ P≤0.05. ${}^{\text{x,y}}$ 0.05<P≤0.10.

### 3.3 Organ measurements

The effects of the organic zinc inclusion rate or the environmental conditions on the internal-organ weight and their percentages relative to the body weight were also calculated and recorded (Table 3). Liver weight was decreased (13 %; P<0.01; Table 3) in 50 HS chicks relative to Ctrl. However, the liver weight as a percentage of BW was decreased (12 %; P<0.01; Table 3) in CHS and 100 HS chicks relative to 50 TN, 100 TN, and 50 HS chicks. There were no treatment differences in heart weight or heart weight as a percentage of BW (Table 3). Intestinal weight was decreased (7 %; P=0.02; Table 3) in HS birds relative to TN birds. Moreover, HS birds tended to have decreased (12 %; P=0.08; Table 3) intestinal weight relative to Ctrl birds. No differences were found in intestinal weight between 50 HS, 100 HS, and Ctrl birds (Table 3), and the intestinal weights relative to BW were similar in all treatments. Compared with Ctrl birds, chicks exposed to HS had a similar spleen weight; however, it was found that spleen weight and spleen weight as a percentage of BW was increased (28 % and 29 %, respectively; P<0.05; Table 3) in 50 HS chicks relative to CHS and 100HS chicks.

**Table 4:** Effects of feeding different levels of organic zinc under TN and HS conditions on intestinal morphology.

Parameter	Treatments						P value	Contrasts		
	Ctrl${}^{1}$	50 TN${}^{2}$	100 TN${}^{3}$	CHS${}^{4}$	50 HS${}^{5}$	100 HS${}^{6}$	Treatment	TN vs. HS	TN vs. TN	HS vs. HS
Duodenum										
Height, µm	758.3 ± 18.0${}^{\text{b}}$	809.6 ± 18.5${}^{\text{a}}$	710.4 ± 20.9${}^{\text{b}}$	739.7 ± 17.8${}^{\text{b}}$	858.3 ± 14.9${}^{\text{a}}$	809.5 ± 22.3${}^{\text{a}}$	<0.01	<0.01	<0.01	0.10
Width, µm	459.9 ± 38.5${}^{\text{ab}}$	357.3 ± 39.4${}^{\text{cd}}$	406.9 ± 44.3${}^{\text{bc}}$	539.3 ± 38.4${}^{\text{a}}$	245.8 ± 40.7${}^{\text{d}}$	486.3 ± 52.4${}^{\text{ab}}$	<0.01	0.65	0.40	<0.01
Depth, µm	202.6 ± 6.3${}^{\text{bc}}$	222.4 ± 6.4${}^{\text{a}}$	210.3 ± 6.0${}^{\text{ab}}$	194.4 ± 5.2${}^{\text{c}}$	221.2 ± 5.3${}^{\text{a}}$	193.5 ± 5.5${}^{\text{c}}$	<0.01	0.07	0.14	<0.01
H : D${}^{7}$	3.86 ± 0.10${}^{\text{b}}$	4.23 ± 0.18${}^{\text{a}}$	3.74 ± 0.13${}^{\text{b}}$	4.00 ± 0.10${}^{\text{ab}}$	3.96 ± 0.09${}^{\text{ab}}$	4.21 ± 0.13${}^{\text{a}}$	0.04	0.25	<0.01	0.18
M index${}^{8}$	3.40 ± 0.10${}^{\text{cd}}$	3.85 ± 0.18${}^{\text{ab}}$	3.77 ± 0.14${}^{\text{b}}$	3.34 ± 0.09${}^{\text{d}}$	4.20 ± 0.13${}^{\text{a}}$	3.75 ± 0.19${}^{\text{bc}}$	<0.01	0.42	0.71	0.03
Jejunum										
Height, µm	687.5 ± 14.8${}^{\text{b}}$	678.8 ± 14.1${}^{\text{b}}$	700.7 ± 21.6${}^{\text{b}}$	625.2 ± 13.3${}^{\text{c}}$	723.8 ± 13.9${}^{\text{b}}$	799.0 ± 18.9${}^{\text{a}}$	<0.01	0.05	0.38	<0.01
Width, µm	187.6 ± 6.4${}^{\text{a}}$	188.5 ± 5.9${}^{\text{a}}$	186.9 ± 6.4${}^{\text{a}}$	163.8 ± 4.0${}^{\text{b}}$	208.0 ± 6.1${}^{\text{a}}$	198.4 ± 14.0${}^{\text{a}}$	0.02	0.73	0.90	0.40
Depth, µm	462.3 ± 35.1${}^{\text{ab}}$	386.6 ± 44.7${}^{\text{bc}}$	357.2 ± 42.4${}^{\text{c}}$	549.2 ± 47.4${}^{\text{a}}$	350.1 ± 35.3${}^{\text{c}}$	343.2 ± 36.4${}^{\text{c}}$	<0.01	0.68	0.67	0.90
H : D	3.94 ± 0.12${}^{\text{b}}$	3.76 ± 0.11${}^{\text{b}}$	3.88 ± 0.12${}^{\text{b}}$	3.93 ± 0.12${}^{\text{b}}$	3.68 ± 0.14${}^{\text{b}}$	4.58 ± 0.18${}^{\text{a}}$	<0.01	0.07	0.54	<0.01
M index	3.46 ± 0.11${}^{\text{bc}}$	3.66 ± 0.14${}^{\text{b}}$	3.75 ± 0.13${}^{\text{b}}$	3.24 ± 0.13${}^{\text{c}}$	3.73 ± 0.12${}^{\text{b}}$	4.33 ± 0.16${}^{\text{a}}$	<0.01	0.19	0.65	<0.01
Ileum										
Height, µm	343.6 ± 5.9${}^{\text{c}}$	393.8 ± 4.3${}^{\text{a}}$	367.6 ± 5.5${}^{\text{b}}$	346.2 ± 5.8${}^{\text{c}}$	402.4 ± 7.7${}^{\text{a}}$	355.4 ± 6.3${}^{\text{bc}}$	<0.01	0.94	<0.01	<0.01
Width, µm	384.8 ± 28.5${}^{\text{a}}$	377.4 ± 27.7${}^{\text{a}}$	239.5 ± 23.2${}^{\text{bc}}$	311.6 ± 31.8${}^{\text{ab}}$	205.3 ± 18.7${}^{\text{c}}$	278.4 ± 24.3${}^{\text{b}}$	<0.01	<0.01	<0.01	0.04
Depth, µm	624.5 ± 23.2${}^{\text{ab}}$	647.3 ± 22.2${}^{\text{a}}$	628.5 ± 25.6${}^{\text{ab}}$	501.8 ± 30.9${}^{\text{c}}$	562.4 ± 28.3${}^{\text{bc}}$	594.6 ± 26.2${}^{\text{ab}}$	<0.01	<0.01	0.60	0.37
H : D	1.94 ± 0.10${}^{\text{d}}$	2.23 ± 0.11${}^{\text{cb}}$	2.40 ± 0.08${}^{\text{b}}$	2.06 ± 0.11${}^{\text{cd}}$	2.78 ± 0.09${}^{\text{a}}$	2.14 ± 0.09${}^{\text{cd}}$	<0.01	0.08	0.21	<0.01
M index	1.81 ± 0.07${}^{\text{c}}$	1.89 ± 0.07${}^{\text{bc}}$	1.90 ± 0.06${}^{\text{bc}}$	2.04 ± 0.08${}^{\text{b}}$	2.29 ± 0.08${}^{\text{a}}$	1.85 ± 0.06${}^{\text{bc}}$	<0.01	<0.01	0.88	<0.01

${}^{1}$ Control chicks under thermoneutral conditions (22.6 ± 1.8 ${}^{\circ }$C; 52 ± 7 % RH) that received 40 ppm of organic Zn. ${}^{2}$ Chicks under thermoneutral conditions (22.6 ± 1.8 ${}^{\circ }$C; 52 ± 7 % RH) supplemented with a diet containing the amount of Zn from the basal diet +50 % of the Zn from the basal diet. ${}^{3}$ Chicks under thermoneutral conditions (22.6 ± 1.8 ${}^{\circ }$C; 52 ± 7 % RH) supplemented with a diet containing the amount of Zn from the basal diet +100 % of the Zn from the basal diet. ${}^{4}$ Chicks exposed to cyclical heat stress (19.8 ± 1.1 ${}^{\circ }$C, 45.3 % relative humidity, from 15:00 to 11:00 LT; 33.7 ± 0.6 ${}^{\circ }$C, 40.9 % RH, from 11:00 to 15:00 LT) on days 36–38 that received 40 ppm of organic Zn. ${}^{5}$ Chicks exposed to cyclical heat stress (19.8 ± 1.1 ${}^{\circ }$C, 45.3 % relative humidity, from 15:00 to 11:00 LT; 33.7 ± 0.6 ${}^{\circ }$C, 40.9 % RH, from 11:00 to 15:00 LT) on days 36–38 supplemented with a diet containing the amount of Zn from the basal diet +50 % of the Zn from the basal diet. ${}^{6}$ Chicks exposed to cyclical heat stress (19.8 ± 1.1 ${}^{\circ }$C, 45.3 % relative humidity, from 15:00 to 11:00 LT; 33.7 ± 0.6 ${}^{\circ }$C, 40.9 % RH, from 11:00 to 15:00 LT) on days 36–38 supplemented with a diet containing the amount of Zn from the basal diet +50 % of the Zn from the basal diet. ${}^{7}$ Villus height to crypt depth ratio. ${}^{8}$ Mucosal surface area is expressed as an M index as described by Kisielinski et al. (2002). ${}^{\text{a,b,c}}$ P≤0.05.

### 3.4 Histological parameters

Compared with TN chicks, HS chicks had decreased villus height 6 % and 3 % in the duodenum and jejunum, respectively (P<0.01; Table 4), while in the ileum, villus height increased by 16 % in 50 HS relative to CHS (P<0.01; Table 4). Villus height in the duodenum was increased by 13 % and 6 % in 50 HS and 100 HS chicks, respectively, relative to the Ctrl chicks (P<0.05; Table 4) and by 16 % and 9 % in 50 HS and 100 HS chicks, respectively, relative to the CHS chicks (P<0.05; Table 3). Similarly, jejunum villus height was increased by 16 % and 28 % in 100 HS chicks relative to the Ctrl and CHS chicks, respectively (P<0.05; Table 4). Duodenum and jejunum villus width in TN and HS were similar. Relative to TN chicks, villus width was decreased by 20 % in HS (P<0.01; Table 4). Mucosal surface area (M index) was increased by 12 % and 34 % in 100 HS chicks relative to the CHS in the duodenum and jejunum, respectively (P<0.05; Table 4). In the ileum, the M index was increased by 12 % in 50 HS relative to CHS (P<0.01; Table 4).

## 4 Discussion

Despite the improvements in heat abatement strategies, HS still compromises farm animal welfare and decreases productivity during the summer. Finding new management strategies will help in ameliorating the negative consequences caused by HS on farm animals. It is now known that HS jeopardizes the intestinal barrier function both directly, due to blood diversion from the internal organs to the periphery, which compromises enterocytes oxygen and nutrient supply, and indirectly, due to hyperthermia-induced hypophagia (Baumgard and Rhoads, 2013), which negatively affects gut health. Zinc supplementation in animal diets has been previously investigated and proved to mitigate and prevent the negative consequences in monogastric models of HS (Pearce et al., 2015; Sanz Fernandez et al., 2014; Sturniolo et al., 2001). Moreover, Pearce et al. (2015) reported that organic zinc (zinc methionine) is more available to farm animals relative to zinc from inorganic sources. Therefore, we hypothesized that a finisher broiler diet supplemented with organic zinc will help in the mitigation of HS effects on broilers at the marketing age by improving the gut health and thus their performance. To our knowledge, the effect of different levels of organic zinc under TN and HS conditions in a finisher diet on broiler chicks has not been investigated in Jordan.

In the current experiment, cyclical HS increased rectal temperature (∼4 %) indicating that we successfully implemented a stressful heat load. As expected, HS decreased fBW relative to the TN chicks (Table 2); it is known that HS has negative effects on the BW of farm animals such as dairy cows (Fabris et al., 2019), sheep (Alhidary et al., 2015), pigs (Seibert et al., 2018; Mayorga et al., 2019), and chickens (Alhenaky et al., 2017); the negative consequences might partially be due to the reduction in feed intake and the diversion of energy towards the presumably activated immune system instead of production purposes (Koch et al., 2019; Mayorga et al., 2019). Interestingly, ADFI were similar in all treatments (P=0.16; Table 2), which contradicts the fact that HS decreases feed intake as indicated previously by Abuajamieh et al. (2018), Johnson et al. (2015), and Mayorga et al. (2019). However, the absence of ADFI differences between treatments in the current study might reflect the short time of heat exposure (4 h for 3 consecutive days) as reported by Marchini et al. (2018). In the current project, FCR was similar in CHS, 100 HS, and Ctrl birds, while FCR in 50 HS chicks was increased relative to the Ctrl birds. Despite treatments tending to have different DPs, the differences found were too small (<5 %) and of a questionable biological importance.

In general, HS caused negative consequences for the morphology of the intestinal tissues, since villus height and M index in the duodenum has numerically decreased relative to TN chicks (Table 4). In the jejunum, HS decreased villus height relative to TN chicks (Table 4). Previous reports have indicated that HS has deleterious effects on intestinal integrity and negatively alters the epithelium of the intestinal tissue (Pearce et al., 2013; Sanz Fernandez et al., 2014). Overall, organic zinc supplementation has improved some morphological structures in the intestinal tissues. In general, 50 HS and 100 HS chicks have increased villus height in the duodenum and jejunum relative to the HS chicks (Table 4). In addition, the epithelial surface area (as indicated by the M index) of the duodenum and jejunum was increased in 50 HS and 100 HS relative to the CHS birds. It was previously reported that organic zinc supplementation improved morphological structures of the intestinal tissues (Pearce et al., 2015; Sanz Fernandez et al., 2014; Shah et al., 2019). The improved gut integrity might be due to the role of zinc in the upregulation of intestinal cell proliferation (Tako et al., 2005).

The results of the organ weights and percentage relative to BW in the current study indicated that HS decreased liver and intestinal weights when compared with TN chicks (Table 3). However, it was reported that liver and intestinal weights were similar in 50 HS, 100 HS, and TN. Previously, it was reported that organic zinc supplementation in animal diets has improved the response of farm animals to HS by improving intestinal architecture and decreasing circulating endotoxins (Sanz Fernandez et al., 2014; Pearce et al., 2015).

As expected, HS chicks were hyperglycemic at the time of blood sampling (right after euthanasia); similar results were found in poultry (Rahimi, 2005) and other farm animals such as pigs (Abuajamieh et al., 2018; Sanz Fernandez et al., 2015), dairy cows (Baumgard et al., 2011), and sheep (Achmadi et al., 1993). Heat stress compromises gut integrity and allows unwanted substances (e.g., endotoxins) to enter the bloodstream (Baumgard and Rhoads, 2013; Abuajamieh et al., 2018). The toxic substances in bloodstream activate the immune system, which is an obligate glucose utilizer (Kvidera et al., 2017; Horst et al., 2018). This might explain the hypoglycemia status right after the HS. Similar results of blood calcium were obtained previously (Mayorga et al., 2019; Kvidera et al., 2017). The decrease in calcium levels found in HS chickens might be explained by increased calcium requirements due to the activated immune system (Hendy and Canaff, 2016; Kvidera et al., 2017).

## 5 Conclusions

Chronic cyclical HS increased rectal temperature, markedly reduced fBW, and decreased productive parameters including ADG and FCR. Herein we demonstrated the organic zinc supplementation at a rate of 100 % of the organic zinc in the basal diet in broilers under HS conditions improved villus height and absorptive surface area (M index) in the duodenum and jejunum and increased the percentage of intestinal weight relative to BW. Moreover, organic zinc supplementation at a rate of 50 % and 100 % of the organic zinc in the basal diet under TN conditions had no effects on the overall performance. Interestingly, dietary organic zinc supplementation in HS chicks increased circulating blood glucose and decreased blood calcium. No other differences were observed with regard to carcass characteristics, body temperature indices, or blood metabolites when chicks were supplemented with organic zinc. However, further studies need to be done using different inclusion rates of organic zinc in order to better understand the different responses of chicks under heat stress conditions.

## Data Availability

The original data are available upon request from the corresponding author.
